# Cranberry/Chondroitin Sulfate Co-precipitate as a New Method for Controlling Urinary Tract Infections

**DOI:** 10.3390/antibiotics12061053

**Published:** 2023-06-15

**Authors:** Concetta Caglioti, Rossana Iannitti, Giada Ceccarelli, Laura Selan, Marco Artini, Rosanna Papa, Antonio Malvasi, Rosaria Gentile, Diletta Del Bianco, Florinda Apone, Paola Angelini, Federico Palazzetti, Bernard Fioretti

**Affiliations:** 1 Department of Chemistry, Biology and Biotechnologies, University of Perugia, Via Elce di Sotto 8, 06132 Perugia, Italy; concetta.caglioti@studenti.unipg.it (C.C.); rosaria.gentile01@universitadipavia.it (R.G.); diletta_delbianco@libero.it (D.D.B.); florinda.apone@studenti.unipg.it (F.A.); paola.angelini@unipg.it (P.A.); federico.palazzetti@unipg.it (F.P.); 2 Department of Medicine and Surgery, Perugia Medical School, University of Perugia, Piazza Lucio Severi 1, 06132 Perugia, Italy; 3 S&R Farmaceutici S.p., Avia dei Pioppi 2, 06083 Bastia Umbra, Italy; r.iannitti@srfarmaceutici.com (R.I.); g.ceccarelli@srfarmaceutici.com (G.C.); 4 Department of Public Health and Infectious Diseases, Sapienza University, Piazzale Aldo Moro 5, 00185 Rome, Italy; laura.selan@uniroma1.it (L.S.); marco.artini@uniroma1.it (M.A.); rosanna.papa@uniroma1.it (R.P.); 5 Department of Biomedical Sciences and Human Oncology, Obstetrics and Gynecology Unit, University of Bari “Aldo Moro”, Piazza Giulio Cesare 11, 70124 Bari, Italy; antoniomalvasi@gmail.com

**Keywords:** cranberry, urinary tract infections, chondroitin sulfate, physical barrier, mucosal protector, hyaluronic acid, *N*-acetylcysteine, bacterial biofilm

## Abstract

Urinary tract infections (UTI), which are among the most frequent cases of infectious diseases, mainly affect women. The most common treatment approach involves the use of antibiotics, although this solution is not always the most suitable, mainly because of the resistance that bacterial strains develop. Proanthocyanidins are a class of polyphenols, abundantly contained in cranberry extracts, which have shown beneficial effects in the treatment of urinary tract infections, due to their anti-adhesive properties toward bacteria, with respect to the membranes of the cells of the urothelium and intestine, thus reducing their virulence. In this work, we demonstrate via microscopy and scattering measurements how a mixture of cranberry and chondroitin sulfate can form a crosslinked structure with barrier properties. By using a design of experiment (DOE), we optimized the mass ratio to obtain a precipitate between cranberry extract and chondroitin sulfate in the presence of N-acetylcysteine and hyaluronic acid. By using transepithelial electrical resistance (TEER) chambers, we confirmed the barrier properties of the best mixture obtained with the DOE. Lastly, the antibiofilm action was investigated against five strains of *Escherichia coli* with different antibiotic sensitivity. The precipitate displayed a variable inhibitory effect in biofilm formation with major effects in UTI with an antibiotic resistance profile.

## 1. Introduction

Urinary tract infections (UTIs) are among the most frequent infectious diseases worldwide [1]. UTIs mainly affect women and patients with diabetes, catheters, prostate infections, immunodeficiencies, and urological abnormalities [2]. UTIs are not life-threatening; nevertheless, they may run into complications and spread to other organs, or lead to recurrence. Indeed, UTIs can be clinically classified into uncomplicated and complicated ones, to distinguish infections of benign origin from those with a high probability of recurrence or progression to more sever forms. The main causative agent of UTIs is *Escherichia coli* (*E. coli*), a bacterium normally present in our intestines, which can colonize extraintestinal niches such as that of the urinary tract via probable anovaginal migration, causing inflammation even with recurrences [3]. Antibiotics are the gold-standard drug treatment for this kind of diseases. Among them, phosphomycin trometamol [4], pivmecillinam [5], nitrofurantoin [6,7], and trimethoprim alone or in combination with a sulfonamide [8] are the first-choice treatments. However, growing antibiotic resistance has negatively impacted UTIs sufferers and the healthcare system [9,10]. Alternatives to antibiotic treatment, especially to prevent recurring UTIs, include cranberry extracts [11,12], D-mannose [13], probiotics [14], vitamin C [15], glycosaminoglycans [16,17], and *N*-acetylcysteine (NAC) [18]. Despite limited clinical data, the use of D-mannose has been shown to have a clinical benefit in the prevention of UTIs [19,20]. D-mannose can competitively inhibit the adhesion of *E. coli* to uroepithelial cells by binding specific glycoprotein receptors [21]. Similarly, the use of glycosaminoglycans (GAGs) such as hyaluronic acid and chondroitin sulfate, orally or via instillation into the bladder, has also become an important tool to strengthen the defense mechanisms of the bladder in patients with recurrent infections [22], supported by clinical evidence [23,24,25]. NAC, a well-known mucolytic agent with a long history of use in the respiratory tract, is also effectively employed in the prevention of UTIs recurrence [17,26,27], by virtue of its antibiofilm properties, which make uropathogens more susceptible to antibiotics [28].

Proanthocyanidins (PACs) are a class of plant-derived molecules contained in various phytocomplexes [29], such as cranberry [30,31], which have demonstrated clinical benefit in the treatment of UTIs. In vitro, PAC-A acts as a bacterial antiadhesive on the membranes of urothelial [30] and intestinal [31] cells, reducing their virulence. To date, considering the low absorption and, therefore, the low urinary concentration [32], many groups believe that the main antiadhesive mechanism of PAC-A on *E. coli*, in vivo, occurs in the intestine [33] rather than in the kidney or in the bladder [32]. Indeed, reducing the ability of *E. coli* strains to colonize the intestine would decrease the possibility of infection while increasing the development of nonvirulent strains [34], paving the way for a possible new scenario in the management of UTIs.

In recent years, it has been shown that xyloglucans isolated from tamarind (*Tamarindus indica*) can form a physical barrier that prevents the interaction of intestinal bacteria with the mucosa, preventing colonization by the pathogens that cause UTIs [35]. In this context, it is not known whether the cranberry in the intestine can create physical antibacterial barriers which, combined with the antiadhesive properties, can contribute to the prophylactic action of UTIs by avoiding the colonization by pathogens.

Considering the increase in antibiotic-resistant pathogens, the search for preventive tools with a nonpharmacological mechanism of action such as nutraceutical and nanomaterials has become of great importance [36,37]. In this context, considering the recent awareness of the central role of intestinal health, we explore the physical barrier coprecipitation approach as a new antibiotic adjuvant against UTIs. The aim of the present work is to understand the best conditions to obtain the coprecipitation that can contribute to the creation of a physical antimicrobial barrier employing known agents used in the treatment of UTIs.

## 2. Results

**Figure 1:**
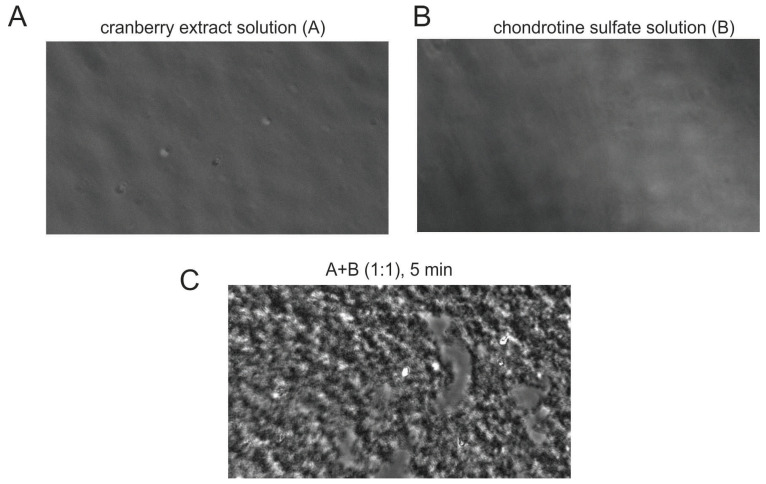
Cranberry/chondroitin sulfate complex formation. (**A**,**B**) Exemplificative microscopic imaging of solutions A and B reported in Table 1. (**C**) Cranberry/chondroitin sulfate complex precipitate obtained by microscopic analysis after 5 min mixture of solutions A and B in 1:1 volume ratio as displayed in Table 2.

First, we studied the precipitates obtained by mixing saturated solutions of substances with antimicrobial action (see Table 1). Since the precipitation of polysaccharide substances is enhanced by the addition of ethanol equal to 30% *v*/*v*, we studied the precipitation process in hydroalcoholic solution. No precipitate was observed in the saturated solutions of the compounds studied (Figure 1A,B; note the absence of precipitate in pure cranberry and chondroitin sulfate solutions at the concentrations shown in Table 1). Surprisingly, when we mixed solutions A and B in a volumetric ratio 1:1 in hydroalcoholic solutions (30%) in the presence of divalent calcium and magnesium ions (see Table 2), we observed the formation of a precipitate (Figure 1C). The divalent ions were added to mimic the intestinal environment [38].

**Figure 2:**
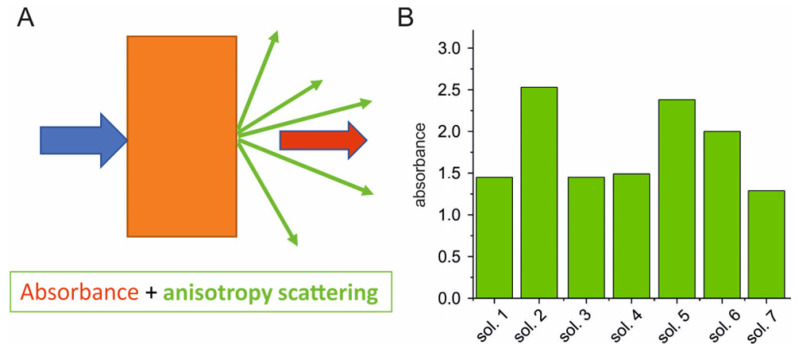
Scatter profile of mixture solutions. (**A**) Schematic view of scatter process observed during spectrophotometric recording. (**B**) Absorption/scattering values for mixtures reported in Table 2 (see Section 2 for description).

The precipitation process was also characterized by evaluating light scattering phenomena in spectrophotometry measurements (see, e.g., [39]). A reduction in absorption of Cranberry solution was observed by dilution. According to microscopic observations, the addition of chondroitin sulfate (solution 2) increased in scattering related to the formation of the suspension (Figure 2). This increase in absorption/scattering was not observed when cranberry was mixed under the same conditions with NAC (solution 3) and hyaluronic acid (solution 4; Figure 2). Other solutions were created according to Table 2, and the scatter process is shown in Figure 2.

**Figure 3:**
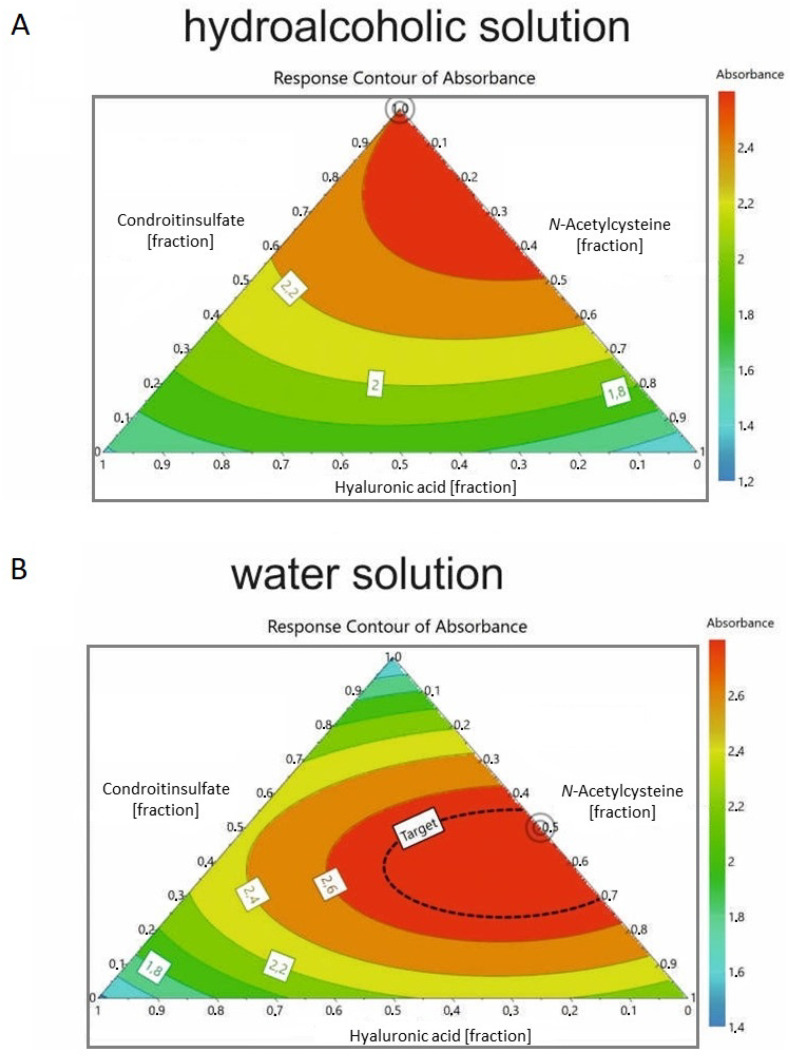
DOE of the precipitation process in (**A**) hydro-alcoholic solution and (**B**) aqueous solution (see Table 2 and Table 3, respectively) obtained by evaluating the scattering process. The double circle represents the best ratio to obtain the scatter process. Colors represent the predicted scattering: blue for low values, green for intermediate values, yellow and orange for intermediate–high values, and red for high values.

The data were analyzed through a DOE approach (see Section 4 “Materials and methods” and Figure 3), highlighting that the best mixture to achieve precipitation in a hydroalcoholic environment was a 1:1 cranberry/chondroitin sulfate ratio, without NAC and hyaluronic acid. The precipitation experiment was extended to an aqueous environment closer to the physiological condition (Table 3) and the results were analyzed with DOE, revealing that the best combination was obtained with the ternary mixture of cranberry/chondroitin/NAC in the ratio 100:50:50 (Figure 3B). Addition of a volume of hyaluronic acid was tolerated, whereas, above 25/200 (*v*/*v*), there was a reduction in precipitation efficiency (solution 15 in Table 3).

**Figure 4:**
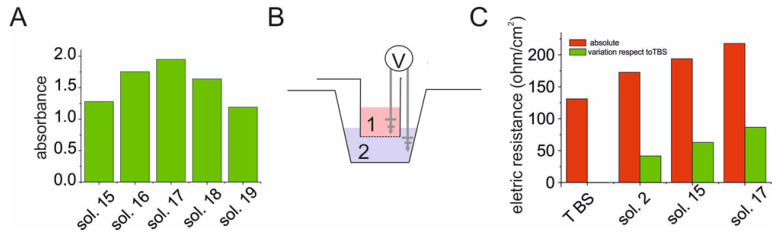
Cranberry *v*/*v* solution ratio impact on precipitation process evaluated by scattering and TEER analysis. (**A**) Optimization of the *v*/*v* ratio of the cranberry solution evaluated with scattering methods for some solutions reported in in Table 3. (**B**) Experimental scheme of the apparatus used in the measurement of the TEER (transepithelial electrical resistance) for the estimation of the electrical resistance of the precipitates. (**C**) Determination of the electrical resistance (Ω/cm^2^) of the precipitate obtained 1 h after mixing the *v*/*v* ratios for the mixtures indicated in Table 2 and Table 3. For TBS, see Section 4 “Materials and methods”.

The optimization process was also explored through the variation of cranberry volume starting from solution 15 of Figure 3, reducing the proportion of CB volume (Figure 4, Table 3). This experiment demonstrated that the best combination of volumes was 60:50:25:25 (solution No. 17 Table 3, Figure 4A). The presence of the precipitate was confirmed by measuring the change in electrical resistance by means of a chamber used to measure the transepithelial electrical resistance (TEER), where the solutions under examination were brought into contact at the interface under the same conditions used for the precipitation process. The electrical resistance of the TBS solution stably measured 148 Ω/cm^2^, while, 60 min after contact with the interface, the electrical resistance increased to 195, 219, and 246 Ω/cm^2^ for solutions 2, 15, and 17 (Figure 4B,C; Table 2 and Table 3) increasing with respect to the TBS solution by 41.5, 62.8, and 86.7 Ω/cm^2^, higher values than those reported with xyloglucans [35].

**Figure 5:**
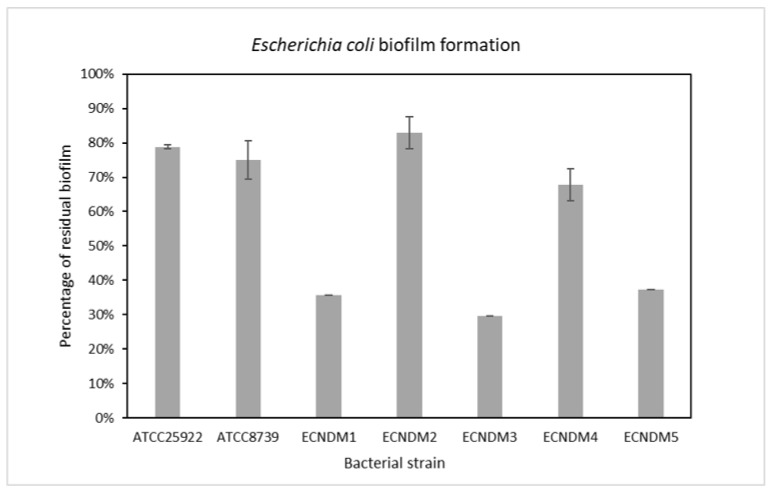
Effect of on biofilm formation of different clinical strains, as well as the ATCC25922 and ATCC8739 reference strains. Cranberry/chondroitin/NAC/hyaluronic acid precipitate was added to the culture medium at time zero (0 h, pre-adhesion period) diluted 1:300, and the biofilm was analyzed after overnight incubation. On the ordinate axis, the bars indicate the percentage of bacterial biofilm production. Data are expressed as the percentage of biofilm formed in the presence of cranberry/chondroitin/NAC/hyaluronic acid precipitate compared with the untreated bacteria. Each data point is composed of three independent experiments, each performed in at least three replicates.

Preliminary experiments were performed to assess the effect of cranberry/chondroitin/NAC/hyaluronic acid precipitate according to solution 17 on the planktonic growth of *E. coli* clinical and reference strains (Table 4). The solutions containing 30% ethanol (Table 2) were not evaluated in order to avoid interference with the antimicrobial action of ethanol. The obtained results showed that the mixture did not affect bacterial growth. Considering these results, biofilm experiments were performed by testing the precipitate according to solution 17 at a dilution of 1:300 for all strains. As a control, bacteria were cultured in BHI medium without the mixture. Results of cranberry/chondroitin/NAC/hyaluronic acid precipitate in the pre-adhesion period are presented in Figure 5. Results are expressed as the percentage of biofilm formed in the presence of cranberry/chondroitin/NAC/hyaluronic acid precipitate compared to the bacteria grown only in BHI medium. Cranberry/chondroitin/NAC/hyaluronic acid precipitate showed an antibiofilm activity on all tested strains, with a percentage inhibition ranging between 70% and 20%. The strongest inhibition (65–70%) was achieved on the isolates ECNDM1 and ECNDM3, representing strains with resistance spread across more classes of antibiotics used in UTIs treatments (Table 4).

## 3. Discussion

One of the most prevalent bacterial diseases worldwide with a significant cause of morbidity is the UTI [1]. Despite the efficacy of antibiotics targeting UTIs, the recurrence rates still remain significant among patients [9], and the development of antibiotic resistance is a major concern, creating a demand for alternative treatment options. Cranberry, *Vaccinium macrocarpon*, is commonly used as a natural alternative preventative treatment for UTIs. Cranberry extracts are composed of vitamin C, organic acids, and polyphenols, including flavonoids and proanthocyanidins. Initially, it was hypothesized that cranberry’s antiseptic action at the urinary level was related to its ability to acidify urine; subsequent experimental studies confirmed that the main mechanism of action depends on inhibiting the adhesion of fimbriated *Escherichia coli* to urothelial cells [40,41], where adhesins allow *Escherichia coli* to adhere to the urothelium according to a ligand–receptor association. In this case, some cranberry components are able to competitively bind bacteria P-type fimbriae, avoiding *Escherichia coli* adhesion to the urothelium. Such components are proanthocyanidins (PAC), a class of polyphenols. PACs can polymerize; according to intermolecular bonding, they are classified into A-type and B-type PACs. B-type PACs are characterized by a single intermolecular bond (C–C) and do not show any antiadhesive bacterial properties. On the contrary, A-type PACs are characterized by both C–C and C–O–C intermolecular bonds. This class of PACs shows antiadhesive bacterial properties, typical of cranberry, which is the fruit richest in proantocyanidins. Recently, it was demonstrated that the antiadhesive properties of the PACs in cranberry are valid for all *Escherichia coli* strains. The following mechanisms were also demonstrated: (i) length and density reduction of P-type fimbriae, together with a reduction in their synthesis; (ii) deformation consisting of elongation of the cell body of the bacterium. Both transformations result in a reduced ability of *Escherichia coli* to adhere to the urothelium. Lastly, a reduction in the adhesion effect also results from changes in the structure properties of bacteria, by a mechanism that involves the electric potential.

In this present study, we reported a new anti-UTI mechanism based on the ability of cranberry extracts to form a precipitate with chondroitin sulfate, in the presence of specific mass ratios with other components such as NAC and hyaluronic acid.

In this context, the precipitate reported herein acts a barrier agent with an intestinal mucosal protective effect [42]. This barrier effect is similar to that of gums, mucilages, glycans, and other substances: In fact, two main mechanisms are involved in the antibiotic action of the intestinal mucosal barrier-protective effect in UTIs. Firstly, by improving the preservation of the physiological intestinal barrier and the tight junctions, the risk of bacterial translocation may be reduced, while not altering the physiological paracellular flow [35]. In fact, the phenomenon of bacterial translocation from the gastrointestinal tract due to an alteration of the intestinal barrier enables bacteria to move to various extraintestinal sites, including the mesenteric lymph node complex, the perineal cavity, the liver, the spleen, the kidneys (therefore, the urinary tract), and the bloodstream, giving rise to secondary diseases and opportunistic infections [43]. A second mechanism is based on antiadhesive and anti-invasive properties, which exert a contrasting action on the formation of the intestinal reservoir of uropathogenes such as *E. coli* species.

In particular, by forming a covering layer on the mucosa, the mucosal barrier prevents the adhesion of *E. coli* (mediated by the fimbriae) and, consequently, the invasion of the epithelium [35,43]. In fact, in spite of excessive intestinal colonization by uropathogens, that by nature tend to adhere, the presence of the protective film prevents their nesting at the epithelial level, favoring their elimination. This second part of the action mechanism is as important as the first one, since accumulation of uropathogens in the intestine with the formation of a bacterial reservoir is an important risk factor for urinary colonization. According to the fecal–perineal–urethral hypothesis, the proximity of the urethra to the terminal tract of the colon is responsible for the external migration of uropathogens and for the contamination of the urinary tract [41]. According to this hypothesis, supported by genetic analyses and clinical evidence, the uropathogens responsible for recurrences in UTI patients are indicated by the intestinal reservoir, highlighting the importance of achieving intestinal eubiosis to maintain urinary tract health [43,44,45].

Uropathogenic *E. coli* contains many virulence factors that confer bacteria resistance to various host defense mechanisms. Recently, it has been shown that, due to the presence of biofilm, bacterial cells are much more resistant to the action of antibiotics and/or immunity cells [46,47]. In fact, bacterial biofilm overgrowth gives bacterial cells both mechanical and metabolic resistance to antimicrobial activities. Mechanical resistance depends on the fact that the polymers, that make up the matrix, limit the diffusion of drugs and other molecules within the biofilm. Metabolic resistance is attributable to the action of β-lactam enzymes produced by the bacteria, which can inactivate antibiotics. Given these characteristics, biofilms can be considered the main cause of antibiotic resistance that complicates the eradication of bacterial infection. In addition, the biofilm is responsible for the continued presence of bacteria in the genito-urethral tract [48]. Furthermore, urinary catheters provide a nidus for infection by serving as a substrate for biofilm formation. Several studies have demonstrated that biofilm cells are more resistant to antimicrobial agents than planktonic bacterial cells [49]. The resistance of biofilms to antibiotics contributes to the persistence of infections, e.g., those associated with implanted devices such as urinary catheters [50].

We are the first to report the ability to obtain precipitates by mixing various components, which are able to adhere to the intestinal lumen and perform a barrier action capable of increasing the permeability of the epithelium. An initial experiment was organized to evaluate the ability of chondroitin sulfate and cranberry to bind and generate a precipitate. It was observed that, when combined in a precise 1:1 ratio, chondroitin sulfate and cranberry interact to form a crosslinked structure. No aggregation is observed if the same components are solubilized in isolation. It was evaluated whether this structure possesses a filtering capacity, such that it can be exploited in a biological context, as a support for a damaged epithelium. To clarify this question, a TEER (transepithelial electrical resistance) experiment was set up, which evaluates the ability of a membrane to oppose the passage of electric current between two solutions. If the barrier manifests electrical resistance, it is, thus, able to filter the invasion and translocation of microorganisms. The electrical resistance of 70 Ω/cm^2^ was seven times higher than that of the xyloglucan barrier [33]. This new mechanism of action exploited by the precise mixture of chondroitin sulfate and cranberry in the presence of specific mass ratios, with other components such as NAC and hyaluronic acid, represents a novel treatment preventative strategy for UTIs given the gut barrier- and mucosal-protective effect, as well as the inhibitory effect on biofilm formation. All these mechanisms promoted by this composition can lead to major effects on UTIs, especially in those with an antibiotic resistance profile.

## 4. Materials and Methods

Preparation of the Solutions. All the components used in this study were dissolved in TBS (composition: TRIS 20 mM, NaCl 150 mM, pH 7.5 with HCl) at the starting concentration defined in Table 1. This concentration expressed in mg/mL is close to the saturation concentration [51,52]. For the cranberry solution, this was obtained by dissolving the 30% commercial extract in proanthocyanidins in TBS until a stable background body was observed. All solutions were centrifuged at 5000 rpm for 10 min. Low-molecular-weight hyaluronic acid, cranberry 30%, bovine chondroitin sulfate, and *N*-acetylcysteine were purchased from Vivatis Pharma.

Microscopic Analysis. Through a light microscope, a drop of the saturated solution, along with the various mixtures (Table 2), was placed on a glass slide, which was cover-slipped and observed under a light microscope at 20× magnification.

Scattering analysis was performed using multiwell ELISA by evaluating the absorption at 550 nm, using the solutions reported in Table 2 and Table 3. Experimental design was performed by Modde^®^ software package (see Modde^®^ 12, Sartorius AG, Göttingen, Germany, User Guide, www.sartorius.com accessed on 8 February 2023). Starting from a defined cranberry solution, the design aimed to identify the ideal composition mixture of chondroitin sulfate, hyaluronic acid, and *N*-acetylcysteine that gives the highest scattering in the ELISA test. The composition values and the related absorbances were optimized according to the D-Optimal design combined with a quadratic model and fitted with a multiple linear regression. These methods gave R^2^ = 0.99 and Q^2^ = 0.81, where R^2^ is the percentage variation of the response explained by the model, while Q^2^ is the percentage variation of the response predicted by the model according to cross-validation. The latter underestimates the goodness of the fit, while the former gives an overestimation. The values of R^2^ and Q^2^ and their difference are best when compared with other combination of designs and models available in Modde^®^.

### 4.1. Bacterial Strains and Growth Conditions

The antibiofilm activity of cranberry/chondroitin/NAC/hyaluronic acid was determined against five clinical isolates of *Escherichia coli* with known resistance profiles and two reference strains belonging to the ATCC collection. Some phenotypic characteristics, such as biofilm formation and antimicrobial profile, of bacterial strains used in this work are summarized in Table 4. All the isolates were retrieved from frozen glycerol stocks, streaked on Brain Heart Infusion agar (BHI, Oxoid, Basingstoke, UK) for 18 h, and sub-cultured to provide fresh colonies. Bacterial cells were grown in planktonic condition at 37 °C under orbital shaking (180 rpm), while biofilm formation was performed at 37 °C in static conditions.

### 4.2. Biofilm Formation

The biofilm content was quantified by microtiter plate (MTP) biofilm assay [53]. Cranberry/chondroitin/NAC/hyaluronic acid (solution 17) was precipitated as previously reported and subsequently deposited on the bottom of each well. The precipitate was used at the following compositions: 1:1, 1:10, 1:30, 1:100, and 1:300. An overnight bacterial suspension was 1:100 diluted into the wells of a sterile 96-well polystyrene flat base plate prefilled with medium in the presence and absence of precipitate. The plates were overnight incubated at 37 °C under static conditions. After incubation, the supernatant containing planktonic cells was gently removed, and the plates were washed. Next, the plates were patted dry in an inverted position. Each well was stained with 0.1% crystal violet for 15 min at room temperature. The excess crystal violet was removed; the plates were washed with double-distilled water and thoroughly dried to quantify the biofilm formation. The adherent biofilm was solubilized with 20% (*v*/*v*) glacial acetic acid and 80% (*v*/*v*) ethanol, and then spectrophotometrically quantified at 590 nm. Each data point was composed of three independent experiments, each performed in at least three replicates.

## 5. Conclusions

A new intestinal mucosal protective agent was described as a consequence of interaction of nutraceutical compounds such as cranberry extract, chondroitin sulfate, NAC, and hyaluronic acid in a specific mass ratio. The biological effects applied to the virulence of UTI pathogens were assessed, and the mixture demonstrated a reduction in biofilm production. On the basis of their effect, the new mucosal-protective agents represent a new potential approach against recurrent cystitis and UTIs that originate from intestinal dysfunction. Further clinical studies are necessary to evaluate the real therapeutic potential of the cranberry extract, chondroitin sulfate, NAC, and hyaluronic co-precipitate in UTIs. The ability to produce physical barriers can be tested in further polysaccharides [54], with a particular interest in β-glucans [55] and GAGs [56] produced by mushrooms.

## Figures and Tables

**Table 1:** Experimental standard solutions.

Solution	Active	Experimental Concentration Solution (mg/mL)
A	Cranberry	15
B	Chondroitin sulfate	50
C	*N*-acetyl cysteine, NAC	100
D	Hyaluronic acid	5

**Table 2:** Solutions employed in the hydro-alcoholic environment. The table reports the composition of the solutions (*v*/*v*) of the following components: A—cranberry, B—chondroitin sulfate, C—NAC, D—hyaluronic acid. The final concentrations of divalent ions in all solutions were 5.5 mM CaCl_2_ and 5.5 mM MgCl_2_.

Solution	EtOH 90%	A	B	C	D
1	200	100			
2	100	100	100		
3	100	100		100	
4	100	100			100
5	100	100	50	50	
6	100	100	50		50
7	100	100		50	50
8	100	100	33	33	33

**Table 3:** Solutions employed in the aqueous environment. The table reports the composition of the solutions (*v*/*v*) of the following components: A—cranberry, B—chondroitin sulfate, C—NAC, D—hyaluronic acid. The final concentrations of divalent ions in all solutions were 5.5 mM CaCl_2_ and 5.5 mM MgCl_2_.

Solutions	A	B	C	D
9	100	33	33	33
10	100	40	30	30
11	100	50	40	10
12	100	50	30	20
13	100	50	20	30
14	100	50	10	40
15	100	50	25	25
16	80	50	25	25
17	60	50	25	25
18	40	50	25	25
19	20	50	25	25

**Table 4:** Antimicrobic properties of cranberry/chondroitin/NAC/hyaluronic acid precipitate. In the first column, bacteria strain codes are reported. Antimicrobial susceptibility was performed according to the guidelines of Clinical Laboratory Standards Institute (CLSI, 2023). AMP: ampicillin; AMC: amoxicillin and clavulanic acid; FOX: cefoxitin; CRO: ceftriaxone; IM: imipenem; ATM: Aztreonam; CIP: ciprofloxacin; AK: amikacin; SXT: trimethoprim/sulfamethoxazole; S: sensitive, R: resistance.

	Penicillins		Cephalosporins		Carbapenem	Monobactams	Fluoroquinolone	Aminoglycoside	Miscellaneous	24 h Biofilm
	AMP	AMC	FOX	CRO	IM	ATM	CIP	AK	SXT	Absorbance
	(10 μg)	(30 μg)	(30 μg)	(30 μg)	(10 μg)	(30 μg)	(5μg)	(30 μg)	(25 μg)	(590 nm)
**ATCC25922**	S	S	S	S	S	S	S	S	S	0.332 ± 0.063
**ATCC8739**	S	S	S	S	S	S	S	S	S	0.230 ± 0.025
**ECNDM1**	R	R	R	R	S	R	R	S	S	0.529 ± 0.236
**ECNDM2**	R	R	R	R	R	R	R	S	R	0.351 ± 0.166
**ECNDM3**	R	R	R	R	S	R	R	S	S	0.175 ± 0.083
**ECNDM4**	R	R	R	R	R	R	R	S	R	0.177 ± 0.087
**ECNDM5**	R	R	R	R	R	R	R	S	R	0.370 ± 0.218

## Data Availability

All data are available in this article.
